# Sorafenib blocks tumour growth, angiogenesis and metastatic potential in preclinical models of osteosarcoma through a mechanism potentially involving the inhibition of ERK1/2, MCL-1 and ezrin pathways

**DOI:** 10.1186/1476-4598-8-118

**Published:** 2009-12-10

**Authors:** Ymera Pignochino, Giovanni Grignani, Giuliana Cavalloni, Manuela Motta, Marta Tapparo, Stefania Bruno, Alessia Bottos, Loretta Gammaitoni, Giorgia Migliardi, Giovanni Camussi, Marco Alberghini, Bruno Torchio, Stefano Ferrari, Federico Bussolino, Franca Fagioli, Piero Picci, Massimo Aglietta

**Affiliations:** 1 Division of Medical Oncology, University of Torino Medical School, A.O. Ordine Mauriziano, Institute for Cancer Research and Treatment Candiolo, Torino, Italy; 2 Unit of Pathology, A.O. Ordine Mauriziano, Torino, Italy; 3 Department of Internal Medicine, Center for Molecular Biotechnology and Center of Experimental Research and Medical Sciences, University of Torino; 4 Division of Molecular Angiogenesis, University of Torino Medical School, Department of oncological science, Institute for Cancer Research and Treatment Candiolo, Torino, Italy; 5 Chemotherapy Division of Department of Musculoskeletal Oncology, Istituti Ortopedici Rizzoli, Bologna, Italy; 6 Division of Pediatric Onco-Hematology, Regina Margherita Children's Hospital, Torino, Italy

## Abstract

**Background:**

Osteosarcoma (OS) is the most common primary bone tumour in children and young adults. Despite improved prognosis, metastatic or relapsed OS remains largely incurable and no significant improvement has been observed in the last 20 years. Therefore, the search for alternative agents in OS is mandatory.

**Results:**

We investigated phospho-ERK 1/2, MCL-1, and phospho-Ezrin/Radixin/Moesin (P-ERM) as potential therapeutic targets in OS. Activation of these pathways was shown by immunohistochemistry in about 70% of cases and in all OS cell lines analyzed. Mutational analysis revealed no activating mutations in KRAS whereas BRAF gene was found to be mutated in 4/30 OS samples from patients. Based on these results we tested the multi-kinase inhibitor sorafenib (BAY 43-9006) in preclinical models of OS. Sorafenib inhibited OS cell line proliferation, induced apoptosis and downregulated P-ERK1/2, MCL-1, and P-ERM in a dose-dependent manner. The dephosphorylation of ERM was not due to ERK inhibition. The downregulation of MCL-1 led to an increase in apoptosis in OS cell lines. In chick embryo chorioallantoic membranes, OS supernatants induced angiogenesis, which was blocked by sorafenib and it was also shown that sorafenib reduced VEGF and MMP2 production. In addition, sorafenib treatment dramatically reduced tumour volume of OS xenografts and lung metastasis in SCID mice.

**Conclusion:**

In conclusion, ERK1/2, MCL-1 and ERM pathways are shown to be active in OS. Sorafenib is able to inhibit their signal transduction, both *in vitro *and *in vivo*, displaying anti-tumoural activity, anti-angiogenic effects, and reducing metastatic colony formation in lungs. These data support the testing of sorafenib as a potential therapeutic option in metastatic or relapsed OS patients unresponsive to standard treatments.

## Background

Osteosarcoma (OS) is the most common primary malignant bone tumour in children and young adults and is characterized by an aggressive clinical course. Chemotherapy significantly increased 5-year survival of localized OS patients to approximately 65% [1]. Pulmonary metastases, central presentation and local non-resectable relapse cause a fatal outcome in the majority of patients [2,3]. Both novel chemotherapeutic drugs and radiometabolic therapy based on samarium failed to improve overall survival [4]. These dismal results are due to P-glycoprotein overexpression [5] as well as complex karyotypes [6], which account for chemoresistance. The search for alternative agents focused on entirely different mechanisms in OS is therefore mandatory.

The advent of molecular targeted therapies has spurred a search for pathological activation of receptors tyrosine kinase (RTKs) via various mechanisms in a number of malignancies including OS. Among the RTKs KIT, Vascular endothelial growth factor receptor (VEGFR) -2, -3 and Platelet derived growth factor (PDGFR)-β have been found to be involved in OS progression and metastatization [7-9].

Two major pathways subsequently activated by RTKs are the phosphatidylinositol 3-kinase (PI3K)/AKT and the mitogen-activated protein kinases ERK 1/2.

Recent studies have demonstrated that the cytoskeletal linker protein, ezrin, a member of the ezrin-radixin-moesin (ERM) family of protein linkers between the actin cytoskeleton and plasma membrane, plays an important role in the metastasis of OS and rhabdomyosarcoma, suggesting that these metastasis-associated molecules could be potential targets for treatment [10]. Matrix metalloproteinases (MMPs) play pivotal roles in tumour invasion through degradation of basement membranes and extracellular matrices [11,12]. MMP-2 and -9 have been found to be involved in OS tumourigenesis and pulmonary metastasization [13,14].

Sorafenib (BAY 43-9006) is an orally active biarylureic multi-kinase inhibitor originally developed to block the ERK 1/2 pathway by targeting Raf-kinases, such as RAF-1 and B-RAF, as well as in the presence of an V600E activating mutation. Off-targets of this drug are other RTKs involved in tumour progression (FLT-3, KIT, fibroblast growth factor receptor, FGFR-1, RET) and angiogenesis (VEGFR-2 and 3, and PDGFR-β) [15]. More recently, it has been demonstrated that sorafenib induces apoptosis in human leukemia cells and other human tumour cell lines through down-regulation of the anti-apoptotic protein myeloid cell leukemia-1 (MCL-1), a Bcl-2 family member [16].

Beyond its preclinical anti-tumoural activity, sorafenib was proven to be effective in 3 different chemorefractory cancers: kidney, liver and thyroid carcinoma. Sorafenib significantly prolongs progression-free survival as well as overall survival of treated patients [17-19].

Several molecular targets of sorafenib seem to be involved in the pathogenesis or progression of OS. One pioneering work demonstrated the amplification of Raf-1 in one case of human OS [20], and the expression of PDGF is associated with OS progression [21]. In addition, VEGF is overexpressed in 63% of untreated OS and is predictive of pulmonary metastasis and poor prognosis [22]. A wide immunohistochemical study on pediatric solid tumours, among them 18 cases of OS, demonstrated that KIT is expressed in the entire case series [7]. Inhibition of the ERK1/2 pathway, mediated by statin treatment, induced apoptosis in OS cell lines [23]. MCL-1 is expressed in a variety of different human sarcoma cell lines, and MCL-1 antisense oligonucleotides combined with low-dose cyclophosphamide provides a synergistic anti-tumour effect, and may qualify as a promising strategy to overcome chemoresistance in human sarcoma [24].

These studies suggest that Sorafenib may be active in OS. However, before exploiting a clinical trial, it was necessary to conduct an investigation of the activation of possible targets of sorafenib in both *in vitro *and *in vivo *models. Thus, we investigated the presence of molecular targets of sorafenib in OS patient specimens and explored the *in vitro *and *in vivo *anti-proliferative effects of this multi-kinase inhibitor as well as its molecular mechanisms of action. In addition, we explored the effect of sorafenib on other pathways potentially involved in progression and metastatic dissemination of OS such as the ERM complex, suggesting a novel sorafenib- targetable molecular pathway.

## Results

### P-ERK1/2, MCL-1 and P-ERM are highly expressed in OS

**Figure 1:**
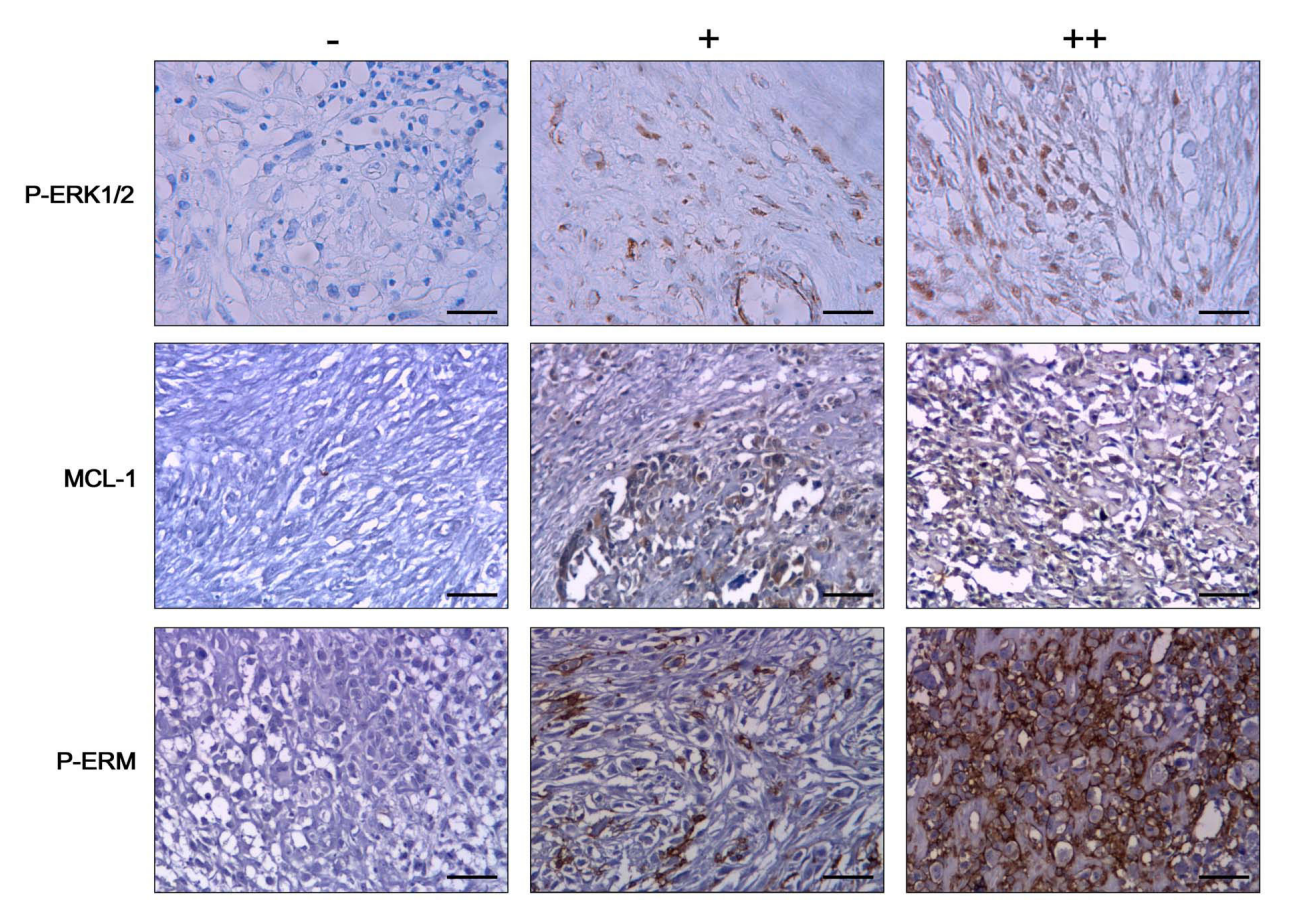
**Representative immunostaining of osteosarcoma samples**. Negative (-), positive (+) and strongly positive (++) samples for P-ERK1/2, MCL-1 and P-ERM expression.

To investigate ERK1/2 pathway activation in OS patients, the expression of phosphorylated ERK1/2 was analyzed in an entire OS case series by immunohistochemistry, and compared with normal adjacent tissues as a control. Nuclear and cytoplasmatic P-ERK1/2 immunostaining was detected in 20 out of 30 samples (66.7%) and 9 of them were strongly positive. Representative examples of P-ERK1/2 staining are shown in Figure 1. These results, demonstrate that the ERK1/2 pathways are activated in all the analyzed histotypes (Table 1).

**Table 1:** Characteristics of OS case series.

Case	Gender	Histology	Grade	Site	BRAF mutations	P-ERK1/2 *	MCL-1 *	P-ERM *
1	M	OSTEOBLASTIC	G4	TIBIA	-	-	+	+

2	M	OSTEOBLASTIC	G4	FEMUR	-	-	+	-

3	F	OSTEOBLASTIC	G4	FEMUR	-	-	-	-

4	M	OSTEOBLASTIC	G4	TIBIA	-	-	-	+

5	M	OSTEOBLASTIC	G4	HOMER	596 delA	-	-	-

6	F	OSTEOBLASTIC	G4	TIBIA	-	-	+	-

7	F	OSTEOBLASTIC	G4	FEMUR	-	-	+	+

8	M	OSTEOBLASTIC	G4	FEMUR	-	-	++	+

9	M	OSTEOBLASTIC	G4	TIBIA	-	-	+	+

10	M	OSTEOBLASTIC	G4	FEMUR	-	-	+	+

11	M	OSTEOBLASTIC	G4	FEMUR	-	++	++	+

12	F	OSTEOBLASTIC	G4	FEMUR	-	+	-	-

13	M	OSTEOBLASTIC	G4	FEMUR	H608L	++	++	+

14	M	OSTEOBLASTIC	G4	FEMUR	-	+	+	-

15	M	OSTEOBLASTIC	G4	HOMER	-	++	++	+

16	M	OSTEOBLASTIC	G4	TIBIA	-	++	+	+

17	F	OSTEOBLASTIC	G4	FEMUR	-	+	+	+

18	M	OSTEOBLASTIC	G4	FEMUR	-	+	++	-

19	F	OSTEOBLASTIC	G4	HOMER	-	++	+	++

20	M	OSTEOBLASTIC	G4	TIBIA	-	+	+	+

21	M	OSTEOBLASTIC	G4	FEMUR	-	++	++	+

22	M	OSTEOBLASTIC	G4	FEMUR	G615R	++	++	++

23	M	FIBROBLASTIC	G4	TIBIA	-	+	++	-

24	M	FIBROBLASTIC	G4	FEMUR	-	++	-	+

25	M	OSTEOBLASTIC	G4	FEMUR	-	+	+	-

26	F	OSTEOBLASTIC	G3	HOMER	-	+	-	+

27	M	OSTEOBLASTIC	G4	FEMUR	-	+	+	++

28	M	OSTEOBLASTIC	G4	FEMUR	S602Y	+	+	+

29	F	OSTEOBLASTIC	G4	HOMER	-	+	++	+

30	M	OSTEOBLASTIC	G4	TIBIA	-	++	++	++

Gender, histological subtypes, grading, site of tumor origin, immunohistochemical results of P-ERK1/2, MCL- 1 and P-ERM expression and BRAF exon 15 mutations of each osteosarcoma samples from patients. intensity of expression: (-)< 10% positive cells scored -, 10-50% positive cells scored +;> 50% positive cells and high staining intensity scored ++).

The normal bone counterpart was consistently negative for activated ERK1/2.

Next, we analysed expression of the MCL-1 protein by immunohistochemistry (Figure 1). Results shown in Table 1 demonstrate that 24 out of 30 (80%) expressed MCL-1 protein in a granular cytoplasmatic staining (mitochondria). Ten out of 24 were strongly positive in more than 50% tumour cells, whilst non-malignant tissues were consistently negative.

The whole series was also analyzed to detect the phosphorylation of cytoskeletal linkers ERM (Figure 1). Twenty-one out of 30 specimens (70%) displayed P-ERM (Table 1) in the cytoplasmatic side of the plasma membrane. In contrast, ERM was not phosphorylated in normal osseous tissues.

Western blot analysis revealed the expression of P-ERK1/2, MCL-1 and P-ERM in the 7 OS cell lines examined (data not shown).

### B-RAF mutations are present in OS samples from patients

The hotspot regions of B-RAF were investigated in the whole series. Exon 15 of B-RAF was mutated in 4 samples (13.3%), as shown in Table 1. One sample had a single-base deletion in codon 596 of the conserved "DFG" motif in the regulatory site. This single base deletion causes a frame-shift that leads to the reading of Val followed by a Stop-codon instead of Gly, and consequently the translation of a truncated form of the protein. A second patient displayed a H608L substitution which has never been described before. A third sample had the G615R mutation. The fourth sample had a point mutation in the activation segment phosphorylation site, causing the substitution of Ser 602 with Tyr (S602Y). These mutations were not presenting in the surrounding non-tumoural tissues. No B-RAF exon 11 and K-RAS exon 1 and 2 mutations were found in the whole case series.

### Sorafenib has anti-proliferative and pro-apoptototic effects on OS cell lines

**Figure 2:**
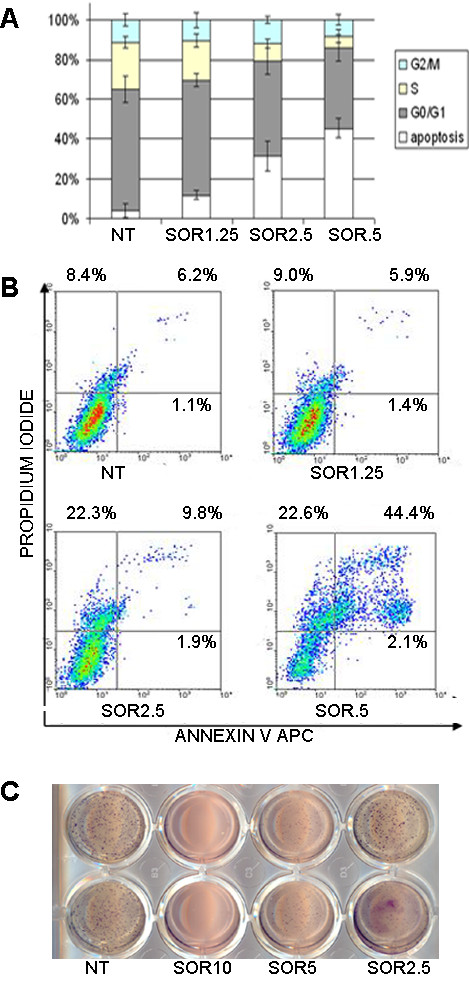
**Sorafenib induces apoptosis and inhibits anchorage independent cell growth of a representative OS cell line**. DNA content (A) and apoptosis (B) analysis after 72 h treatments. Soft agar assays as described in "materials and methods" section (C).

To investigate the effects of sorafenib on *in vitro *proliferation, we exposed 7 different OS cell lines to increasing doses of the drug for 24, 48 and 72 hours. CellGlo™ assays demonstrated that sorafenib caused a dose- and time- dependent cell growth inhibition of all the 7 cell lines tested. IC50 values after 72 hours of treatment were calculated on the basis of these results and are shown in Table 2. At this time point, DNA content and apoptosis analysis was evaluated by FACS. Sorafenib did not induce cell cycle arrest, but a dose dependent increase of the percentage of cells in sub-G_0 _phase considered to be apoptotic cells (Figure 2, panel A). Further Annexin V/PI staining confirmed that sorafenib induced a dose-dependent increase in the percentage of apoptotic cells, as shown in Figure 2, panel B. In addition, sorafenib displayed a dose-dependent inhibition of anchorage-independent cell growth, as shown by soft agar assays (Figure 2, panel C).

**Table 2:** Concentrations of sorafenib inhibiting 50% of OS cell line proliferation.

Cell line	IC50 (μM)	Confidence Intervals (μM)
MG63	2.793	2.32-3.35

U2OS	4.677	2.99-6.61

SAOS-2	3.943	1.87-5.31

KHOS	2.417	1.88-3.10

HOS	4.784	3.99-5.48

SJSA-1	4.511	3.01-6.71

MNNG-HOS	4.814	3.13-6.39

IC_50 _values (μM) and relative 95% confidence intervals obtained after 72 hours of sorafenib exposure.* IC50: Sorafenib concentration inhibiting 50% of the cell line proliferation.

### Sorafenib down-regulates P-ERK 1/2, MCL-1 and P-ERM expression in OS cell lines

**Figure 3:**
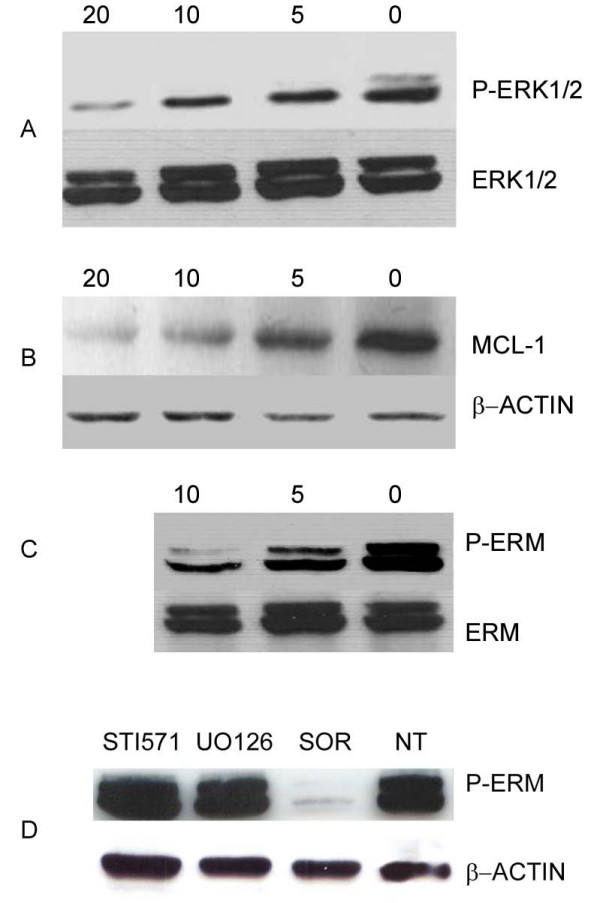
**Sorafenib induces down-regulation of P-ERK 1/2 (A), MCL-1 (B) and P-ERM (C) on U2OS**. Effect on P-ERM is independent from PDGFR, C-KIT and ERK pathways (D). Cells were cultured for 24 hours with escalating doses (5, 10, 20 μM) of sorafenib, 10 μM STI571, 10 μM UO126 or left untreated. After protein extraction P-ERK 1/2 (A), MCL-1, (B), P-ERM (C and D) immunoblotting was carried out.

To elucidate the mechanisms of cell growth inhibition and apoptosis induced by sorafenib, OS cells were exposed to the drug at concentrations ranging from 0 to 20 μM for 24 hours. Results demonstrated that sorafenib induced a dose-dependent decrease in phosphorylated ERK1/2 and ERM in all the 7 cell lines tested. Representative western blots are shown in Figure 3 (panel A and C). Expression of total ERK and ERM was not affected by sorafenib treatment.

To verify whether ERM phosphorylation is dependent on PDGFR or KIT pathways, OS cell lines were treated with imatinib mesylate (STI571, Gleevec) a known inhibitor of PDGFR and KIT as well as ABL. As shown in Figure 3 (panel D) STI571 treatment did not affect ERM phosphorylation.

In addition, the effect of sorafenib on phosphorylation of ERM is not ERK dependent. Indeed, the inhibition of ERK pathway resulting from treatment with UO126, a MEK-specific inhibitor, did not affect phosphorylation of ERM (Figure 3, panel D).

The expression of MCL-1 in OS cells treated with sorafenib for 24 hours was analyzed by immunoblotting. A significant dose-dependent reduction of MCL-1 protein was detected (Figure 3, panel B).

### Inhibition of MCL-1 expression induces apoptosis in OS cell lines

**Figure 4:**
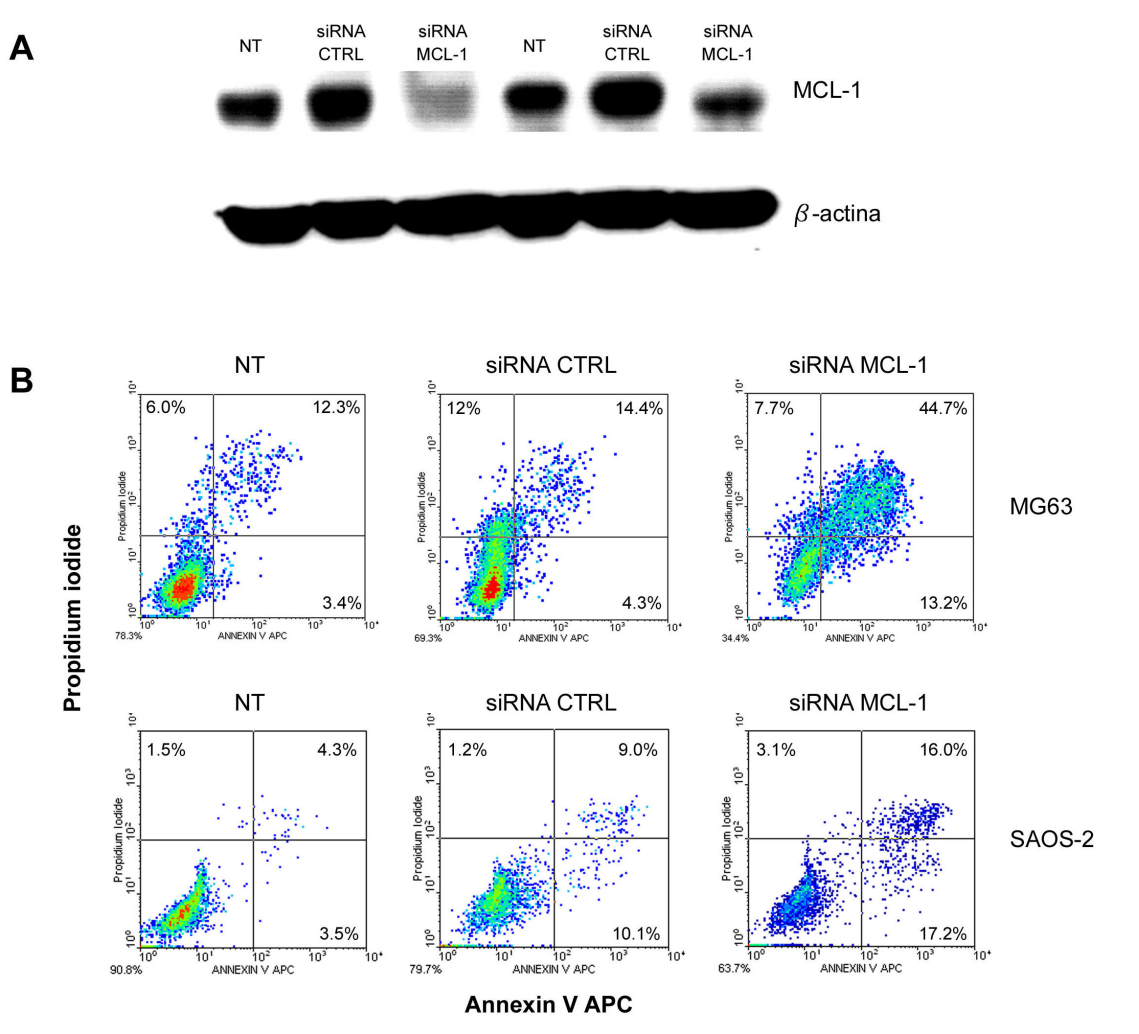
**MCL-1 down-regulation induces apoptosis in OS cell lines**. MCL-1 protein expression (A) and annexin V/PI staining (B) in MG63 and SAOS-2 after MCL-1 silencing with siRNA.

In order to investigate if the anti-apoptotic effect of sorafenib could be attributable to the inhibition of MCL-1 we exploited siRNA technology. SiRNA MCL-1 transfection significantly decreased MCL-1 protein expression in all the 7 cell lines tested. Different OS cell lines displayed different sensitivity to MCL-1 silencing. Namely, in MG63 cells, which were the most sensitive to MCL-1 silencing, there was a strong reduction in MCL-1 protein expression, as demonstrated by western blot analysis (Figure 4, panel A). Meanwhile, in SAOS-2 cells, the least sensitive to MCL-1 silencing, only a minor down-regulation of MCL-1 protein was observed (Figure 4 panel A). SiRNA-induced MCL-1 down-regulation produced an increase of apoptotic OS cells compared to cells transfected with control siRNAs (Figure 4, panel B). The percentage of late apoptotic cells was higher in MG63 cells than in SAOS-2 cells (44.2% vs 16.0% respectively), reflecting the level of MCL-1 down-regulation.

### Sorafenib inhibits MMP2 and VEGF production in OS cell lines

**Figure 5:**
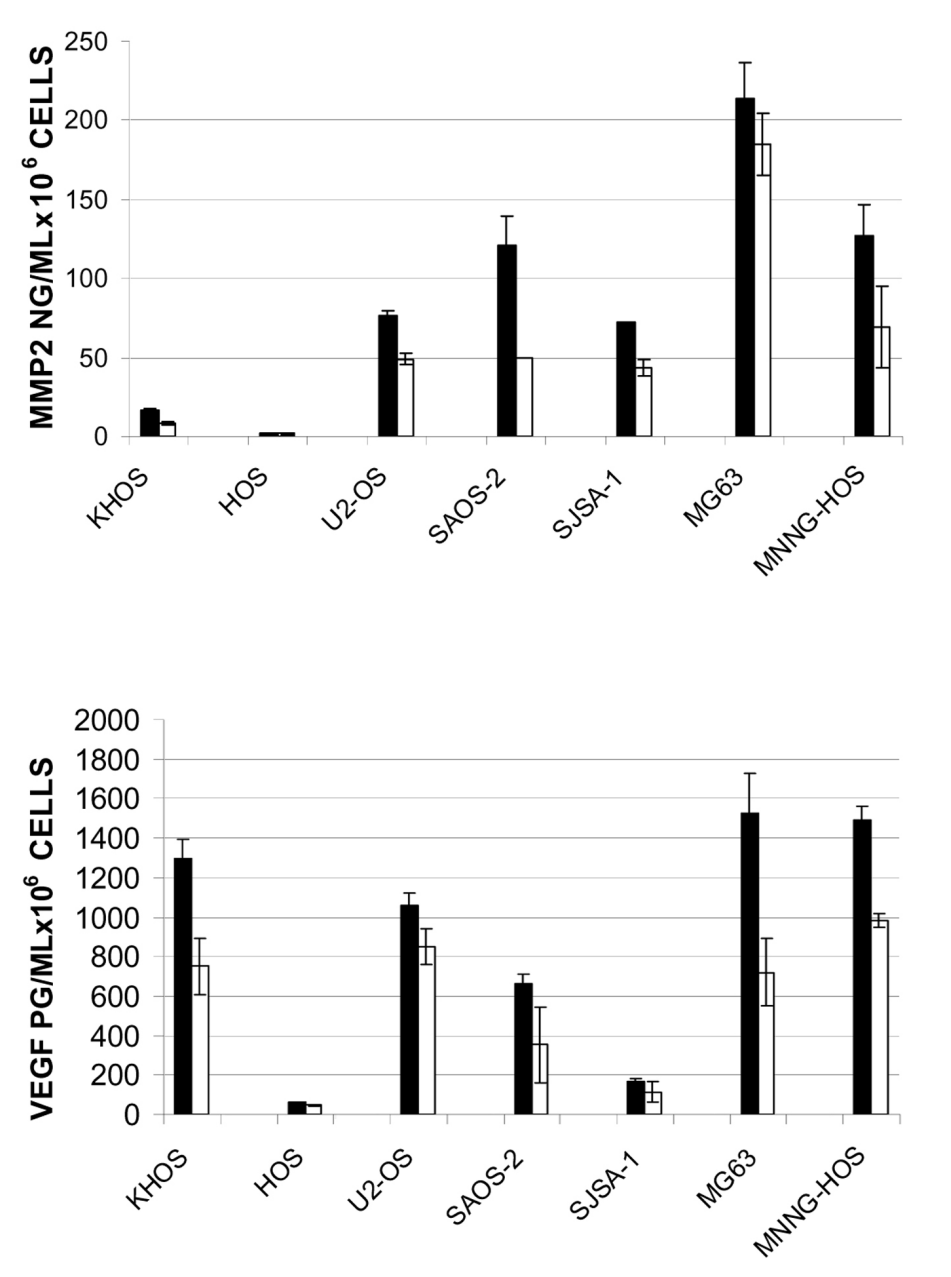
**ELISA test for MMP2 and VEGF quantification on osteosarcoma cell line supernatants**. The concentration of secreted MMP2 (a) and VEGF (B) on 48 h conditioned supernatants of untreated (Black bars) and 5 μM sorafenib treated (white bars) OS cells was shown per 10^6 ^cells as Mean +/- standard deviation (Y error bars) of 2 different experiments in triplicate. a, *p *< 0.05 versus untreated cells; b, *p *< 0.05 versus sorafenib.

To explore the activity of sorafenib on the effectors involved in tumour progression and angiogenesis, we measured MMP2 and VEGF production in supernatants of all the 7 cell lines tested. We observed that different cell lines exhibit different basal level of MMP2 and VEGF-A, being higher in MG63 cells, and lower in HOS cells (Figure 5). Treatment with sorafenib produced a consistent reduction of the concentration of MMP2 and VEGF-A in all cell lines tested (Figure 5). However, the magnitude of this reduction was heterogeneous. Namely, after 48 hours MMP2 produced by 10^6 ^cells was reduced to 47.8% in KHOS, 64.8% in HOS, 63.9% in U2-OS, 40.7% in SAOS-2, 59.6% in SJSA-1; 86.5% in MG63; and 54.4% in MNNG-HOS cells.

Sorafenib treatment led to the reduction of VEGF-A produced by 10^6 ^cells to 57.7% in KHOS, 73.1% in HOS, 80.5% in U2-OS, 52.9% in SAOS-2, 67.5% in SJSA-1; 47.1% in MG63; and 65.7% in MNNG-HOS cells.

### Sorafenib has an anti-angiogenic effect in CAM

Chick chorioallantoic membrane assay was performed to investigate the angiogenic potential of OS cell lines and the anti-angiogenic effect of sorafenib *in vivo*.

**Figure 6:**
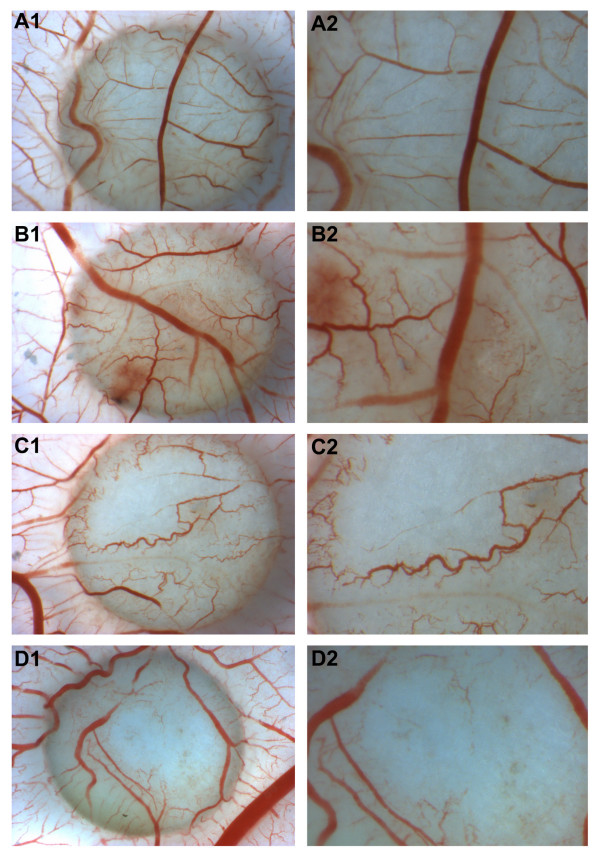
**Sorafenib inhibits angiogenesis in CAM mode**. A. Culture medium (n = 4). A1: 10× magnification, A2: 20× magnification; B. Osteosarcoma cell line conditioned medium (n = 6). B1:10× magnification, B2: 20× magnification; C. Conditioned medium of sorafenib treated cells (n = 5). C1:10× magnification, C2: 20× magnification; D. Osteosarcoma conditioned medium plus sorafenib 1 μM (n = 7). D1:10× magnification, D2: 20× magnification.

The supernatant of U2OS cells clearly increased sprouting angiogenesis in CAM (Figure 6, panel B) compared with culture medium alone (Figure 6, panel A), indicating the secretion of angiogenic factors by OS cells. Anti-angiogenic effects of sorafenib were tested by two different approaches, i.e. treating the cells prior to CAM stimulation or directly adding sorafenib into the CAM already stimulated with untreated tumour cell supernatant. When U2OS were treated with low concentration of sorafenib to avoid cell mortality,, the supernatant developed a lower angiogenic response than untreated cells (Figure 6, panel C), probably due to the decrease of secreted angiogenic factors. The treatment of CAM with sorafenib blocked angiogenesis induced by U2OS cell supernatant, suggesting that the drug may also act on host vasculature (Figure 6, panel D).

### Sorafenib displays anti-tumoural activity in vivo against human OS xenografts

**Figure 7:**
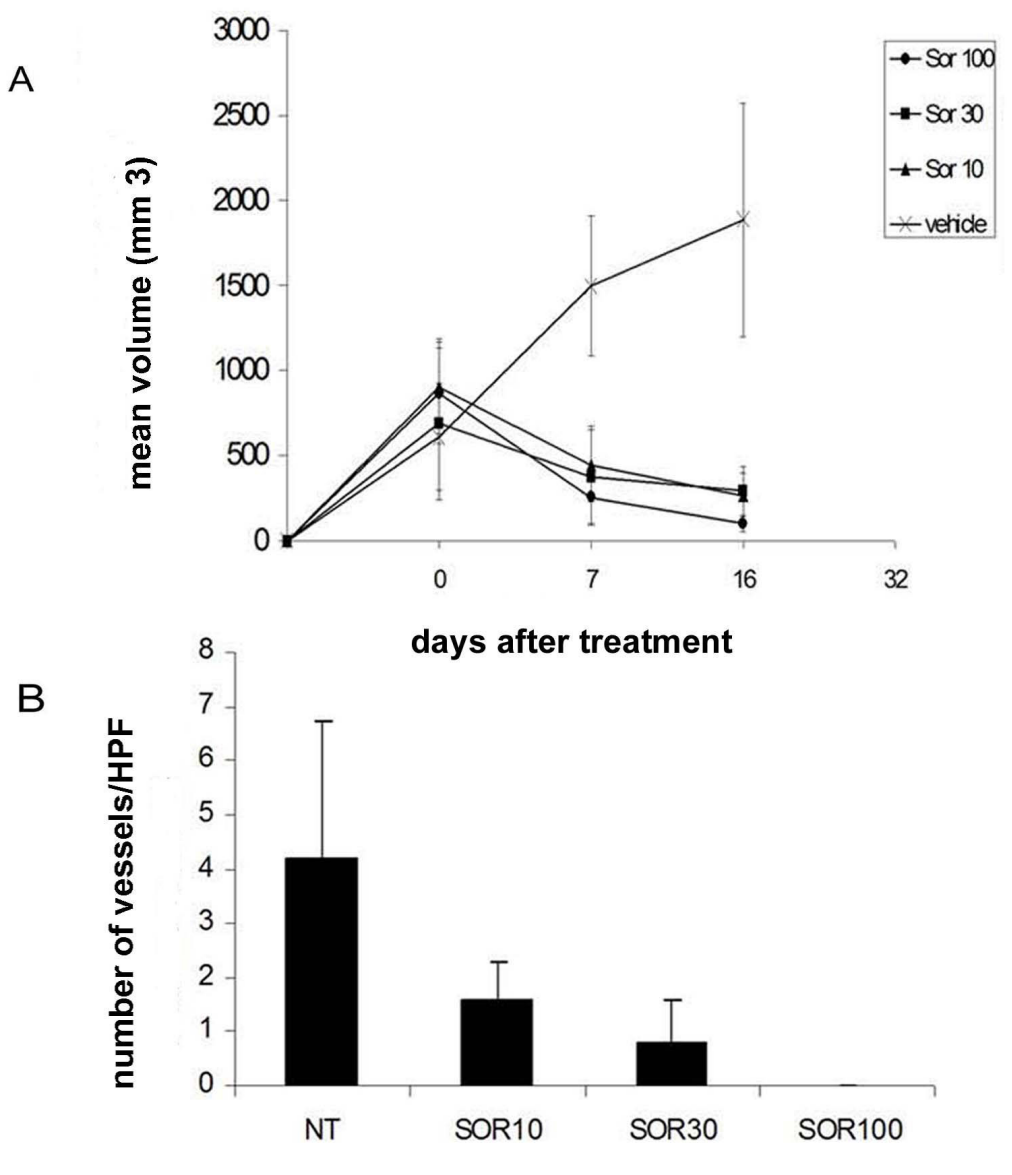
**Sorafenib activity in OS xenograft models**. In three different experiments, one group of mice was injected s.c. into the right flank with U20S and one group of mice was injected e.v. with SJSA-1 cells. Five mice per group were orally treated daily with sorafenib (0-10-30-100 mg/kg/die) for 16 days as described in the Materials and Methods section. (A) Sorafenib reduces tumour volume OS xenografts in SCID mice. The graph indicates the mean tumour volume (mm^3^) measured using calipers at 0, 7 and 16 days of sorafenib treatment (Error bars: standard deviation). (B) Sorafenib reduces blood vessel number. The graph shows the mean number of patented blood vessel +/- standard deviation calculated in 10 fields at 40× magnification on Masson's trichromic staining.

Based on their median level of MMP2 and VEGF-A production, and their previously demonstrated tumourigenicity in mice [14], U2OS and SJSA-1 cell lines were chosen for *in vivo *studies. Sorafenib treatment dramatically reduced tumour volume of s.c. U2OS xenografts in SCID mice compared to untreated mice as shown in Figure 7 (Panel A). Moreover, the number of patented blood vessels was strikingly reduced in tumours of treated mice, as shown in Masson trichromic stained sections (Figure 7, panel B).

**Figure 8:**
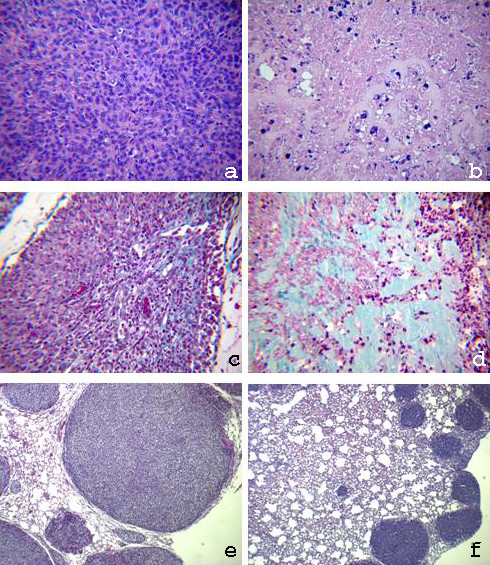
**Sorafenib reduces tumour mass in OS xenografts in SCID mice**. Histological sections derived from sorafenib untreated (a) and treated (b) xenografts stained with Hematoxylin and Eosin show massive necrosis and reduced cellularity. Sorafenib reduces blood vessel number. 40× magnification on Masson's trichromic staining of sorafenib untreated (c) and treated (d) xenografts. Sorafenib reduces lung OS foci. Pictures show representative examples (10× magnification) of OS foci in lung sections of untreated (e) and sorafenib-treated mice (f).

Histological analysis revealed that sorafenib-treated xenografts had a lower tumour cell number, which mostly showed marked regressive nuclear changes as pyknosis (Figure 8, panels A-B). In treated mice, OS viable cells were present on the edge of the lesion showing a generalized shrinkage of the viable tissue thickness. A sharp increase in the extracellular matrix is evident in histological sections from sorafenib-treated mice compared to non-treated mice (Figure 8, panel C-D).

### Sorafenib reduces metastasis formation in lungs

One million SJSA-1 cells were injected into the tail vein of SCID mice giving rise to pulmonary colonies within 3 weeks. Subsequent treatment with sorafenib for 16 days inhibited tumour colony growth. In some of the treated mice with the highest sorafenib dosage, a massive lung collapse with pulmonary bleeding was observed at autopsy. The percentage of area occupied by lung foci analyzed per optical field after hematoxylin and eosin staining at lower magnification (40×) was 73% ± 14, 84% ± 11,40% and 35% ± 7 in sorafenib 10, 30, 100 mg/kg/day treated mice respectively. In Figure 8, panels E-F, representative sections of lung from untreated- and sorafenib-treated mice are shown.

### Sorafenib down-regulates P-ERK 1/2, MCL-1 and P-ERM expression on OS xenografts

Molecular targets of sorafenib were also evaluated in xenograft sections. Immunohistochemical analysis clearly demonstrated the reduced phosphorylation of ERK1/2, with the percentage of positive cells diminishing from 84% (score ++) in sections of untreated xenografts to 15%, 30% (score +) and 6% (score -) in 10, 30 e 100 mg/kg/die sorafenib treated mice respectively (p < 0.05). Expression of MCL-1 is also slightly down-regulated following sorafenib treatment. MCL-1 was expressed in 90% cells (scored ++) of untreated xenografts and in 75% (++), 60% (++) and 40% (+) in 10, 30 and 100 mg/kg/die sorafenib-treated mice respectively (p < 0.05). P-ERM is strongly expressed along the inner layer of the plasma membrane in a continuous fashion in 50% of viable cells in untreated mice (scored ++). Instead, sections from treated xenografts displayed a weak P-ERM staining in a discontinuous fashion (scored +).

## Discussion

It is well known that ERK 1/2 play a key role in different neoplasia. Moreover, even in cancer like GISTs driven by other kinases, activation of KIT or PDGFR-α lead to phosphorylation of ERK 1/2 [25] and these kinases mediate the proliferative advantage of the neoplastic cells. Imatinib blocks KIT signalling, causing the proliferative arrest of gastrointestinal stromal tumours (GISTs), and the inactivation of ERK 1/2. However, resistance to imatinib is accompanied by reactivation (phosphorylation) of ERK 1/2. Among the new drugs with specific molecular targets sorafenib was shown to be effective in renal cell carcinoma and hepatocarcinoma, I through the inhibition of ERK1/2 pathway [17,18]. These results, along with the unsatisfactory outcome of relapsed and metastatic OS cases led us to investigate the presence and role of sorafenib targets in paraffin-embedded tissue from OS patients as well as in several OS cell lines and, thereafter, to explore sorafenib activity in xenograft models of human OS.

We demonstrated that 66.6% of OS samples from patients displayed an activated ERK 1/2 pathway suggesting that it may be relevant in increased OS proliferation. There was only one prior datum [26] addressing the overexpression of ERK 1/2 in OS. We observed a highly reproducible and consistent expression of P-ERK1/2 among OS specimens. Furthermore, activated ERK 1/2 were only present in the neoplastic tissue and not in the normal tissue surrounding the tumour. Its selective expression is a clue to a proliferative role compared to normal tissue.

Another metabolic pathway often involved in tumour growth advantage regards the mechanisms preventing apoptosis. Among sorafenib off-targets, we investigated and found the antiapoptotic protein MCL-1 activated in 84% of OS specimens, emphasizing its role as a possible mechanism of survival after chemotherapy. This result is intriguing as it may represent both a reason to test sorafenib activity independently from ERK 1/2 expression and a possible future target itself in OS.

Mutational status of the target is critical to the effective inhibition by small inhibitors in at least two cancers: non small cell lung cancers [27] and GIST [25]. Constitutive ERK activation is common in human cancers and is often the result of activating mutations of B-RAF and K-RAS. A B-RAF mutation occur in approximately 8% of human tumours (melanoma, papillary thyroid cancer and colon cancer) and in over 80% of cases it is represented by a single base-pair substitution in exon 15 at codon 600 (V600E) [28]. Therefore, we evaluated the presence of B-RAF mutations in our series. We demonstrated B-RAF mutations in 13.3% of cases: three mutations have already been described, whilst H608L is a novel point mutation of unknown functional significance. On the contrary, we could not detect any mutations in B-RAF exon 11 or in K-RAS exon 1 and 2. Since sorafenib is active in wild type B-RAF, and mutated forms represent only a minority, this finding does not stand against its clinical application in OS.

Ezrin was recently pointed out as one of the major determinants of metastatic behaviour in OS [29]. We investigated the expression of active ERM complexes (the phosphorylated form) in order to show if the ezrin pathway was active in the OS case series examined. Interestingly, for the first time, we showed ERM activation in 70% of cases and in all the OS cell lines tested.

Our data strengthen the role of ezrin in OS and the need to further explore the targeting of ezrin in this neoplasia.

*In vitro *preclinical models of human OS cell lines allowed us to test sorafenib activity. All the 7 cell lines we studied clearly showed that sorafenib inhibits OS cell growth. This event is not due to cell cycle arrest but to the induction of apoptosis, probably via a mechanism involving the MCL-1 downregulation, as already demonstrated in acute myeloid leukemia [16]. Indeed, MCL-1 silencing with specific siRNA induced an increase of apoptotic cells in OS in vitro models.

Furthermore, sorafenib activity in OS could be mediated by P-ERK 1/2 and P-ERM downregulation involved in proliferation and metastasization respectively [15,29].

Since the UO126-induced inhibition of the ERK pathway does not affect ERM phosphorylation we can affirm that sorafenib is able to down-regulate signalling through ERM in an ERK-independent manner. This effect is also PDGFR-independent. Indeed, treatment of OS cell lines with STI571 does not change the phosphorylation status of ERM. Our findings unveiled the ERM pathway to be a novel molecular target of sorafenib, and prompted us to further investigate this molecular mechanism of action.

Matrix metalloproteinases are one of the main causes of the invasive phenotype of tumour cells. It is noteworthy that MMP2 (gelatinase A) has been implicated in invasion and metastasis in several cancers [10,11]. We demonstrated that sorafenib is able to inhibit MMP 2 production by OS cell lines, consistent with ERK1/2 involvement in the induction of MMPs [30,31]. Furthermore, the reduction of MMP2 production may determine a diminished invasiveness potential of OS. This finding is an intriguing aspect of sorafenib use in the clinical setting of OS.

VEGF, the principal stimulator of angiogenesis, is also involved in the metastatic behaviour of OS [9]. We showed sorafenib induces a consistent reduction of VEGF production in OS cell lines, most likely due to ERK1/2 inhibition. Indeed, VEGF mRNA was blocked by the ERK1/2 pathway inhibition [32].

Therefore, the anti-tumoural activity of sorafenib in OS may also be caused by inhibition of the blood supply due to the reduction of new blood vessel formation, as observed in CAM assays, confirming its antiangiogenic activity.

A xenograft OS model allowed us to verify whether sorafenib would modify the growth of OS cell lines *in vivo*. Our results clearly show sorafenib had a major impact on this endpoint. OS cell lines inoculated in SCID mice grow at a very high rate, causing death of the recipients in a short time. Sorafenib strongly reduced tumour dimensions after 16 days of treatment even at a lower dosage (10 mg/Kg/die). Two aspects must be stressed: sorafenib treatment began with established masses, just as in human OS relapses where tumours are also often dimensionally conspicuous. Secondly, we observed significant tumour shrinkage after a relatively short course of therapy. This is expected to be the usual response to chemotherapy drugs, but not necessarily to small inhibitors as TK inhibitors may be efficient in prolonging survival without any significant tumour shrinkage [33]. Dimensional tumour response might imply a major antitumour effect of this drug in OS. Finally, lungs are by far the most frequent metastatic site in OS. In our xenograft model, Sorafenib was shown to reduce mouse death rate and we demonstrated a reduction in the dimension and number of lung nodules. On OS xenografts, immunohistochemistry analysis revealed that ERK1/2, MCL-1 and ERM were consistently inhibited, confirming the sorafenib-induced mechanisms of action. As in renal cell carcinoma and in hepatocarcinoma, the antiangiogenic properties of sorafenib may play a major role in its anti-tumoural effect in OS as well. However, elevated VEGF production is not a requirement for sorafenib activity in OS, since sorafenib was also effective in the SJSA-1 xenografts which produce lower levels of VEGF compared to other OS cell lines.

## Conclusion

Due to the discouraging results of present therapies in relapsed OS, our work was primarily focused on searching for molecular cues useful for new therapeutic approaches as target therapies. We identified a consistent expression of activated ERK1/2, MCL-1 in a homogeneous OS case series. These molecular players represent suitable targets of sorafenib. In particular, sorafenib caused *in vitro *and *in vivo *down-regulation of MCL-1 and inhibition of the ERK1/2 pathway. For the first time, we demonstrated that ERM, a well known marker of tumour progression and metastasis, was largely expressed in OS specimens, and that sorafenib inhibited its phosphorylation in *in vitro *and *in vivo *models. Lastly, we demonstrated an *in vitro *pro-apoptotic effect of sorafenib and an anti-tumour activity in OS xenograft in murine models. We believe these data support an investigation of sorafenib activity in a phase II study in relapsed or unresectable metastatic patients affected by OS following the failure of conventional therapies.

## Methods

### OS specimens and cell lines

A homogeneous case series (Table 1) of formalin-fixed paraffin-embedded samples of 27 osteoblastic osteosarcoma grade G4, 1 osteoblastic osteosarcoma grade G3 and 2 fibroblastic osteosarcoma grade G4 were collected at the Istituti Ortopedici Rizzoli, Bologna, Italy.

Seven osteosarcoma cell lines (SJSA-1, U2OS, SAOS2, MG63, HOS, MNNG/HOS and KHOS) were purchased from American Type Culture Collection (Manassas, VA, USA). All cells were cultured in RPMI 1640, (Invitrogen, Paisley, UK) in the presence of 10% heat inactivated fetal calf serum (FCS), 1% L-glutamine, and penicillin/streptomycin (10,000 U/ml and 10,000 g/ml, respectively), with medium changes every 2 to 3 days.

### Immunohistochemistry

The expression of phospho-ERK1/2 (P-ERK1,2), MCL-1 and P-ERM proteins was performed on paraffin-embedded tumour sections (5 μm) mounted onto ChemMate Capillary Gap Microscope slides (Dako Cytomation, Milan, Italy), dried in a 45°C oven for 12 hours, deparaffinized in xylene, and rehydrated in graded alcohols and distilled water. Sections were heated in 10 mM citrate buffer pH 6.0 in a water bath at 96°C for 45 minutes, cooled, and stored in TBS at pH 7.6. Endogenous peroxidase activity was blocked with 0.3% hydrogen peroxide for 10 minutes, followed by treatment with V-block (Lab Vision Corporation, Fremont, CA, USA) for 30 minutes. Sections were incubated overnight in a moist chamber at 4°C with the primary antibodies anti MCL-1 (MAB4602 dilution 1:100 Chemicon-Millipore, Temecula, CA), anti P-ERK1/2 and anti P-ERM (20G11 and 41A3 dilution 1:100 Cell Signaling Technologies, Danvers, MA, USA). After washing in TBS-Tween, sections were incubated with secondary antibody and horseradish peroxidase conjugated with polymer for 30 minutes. Staining was visualized using 3-3'diaminobenzidine (Envision+ Dual Link System-HRP, Dako Carpinteria, CA, USA) for 5 minutes, counterstained with Mayer's hematoxylin (Sigma Chemicals, St. Louis, MO, USA) for 1 minute, dehydrated in a series of graded ethanol, cleared in xylene and mounted. Eureka imaging technology was used to analyze 1000 cells per sample. Staining intensity as well as the percentage of maximally stained tumour cells in each core biopsy were recorded (< 10% positive cells scored -, 10-50% positive cells scored +; > 50% positive cells and high staining intensity scored ++).

### Mutational analysis

Genomic DNA was extracted from deparaffinized samples and cell lines, with the use of the QIAamp DNA Mini Kit (Qiagen, Milan, Italy) following the manufacturer's instructions. To search more frequent mutated sites, exon 1 (forward primer: GAATGGTCCTGCACCAGTAA; reverse primer: GTGTGACATGTTCTAATATAGTCA) and exon 2 (forward primer: GGTGGAGTATTTGATAGTGTATTAACC reverse primer AGAATGGTCCTGCACCAGTAA) of K-RAS, exon 11 (forward primer TCCCTCTCAGGCATAAGGTAA; reverse primer: ATCAAAGGAAATATTCACTGTTCG) and exon 15 (forward primer: TGCTTGCTCTGATAGGAAAATG; reverse primer: AGCATCTCAGGGCCAAAAAT) of B-RAF were amplified by PCR.

PCR products were then purified using QIAquick PCR purification kit (Qiagen Milan, Italy) and sense and antisense sequences were obtained by using forward and reverse internal primers respectively. Each exon was sequenced using the BigDye Terminator Cycle sequence following the PE Applied Biosystem strategy and Applied Biosystems ABI PRISM3100 DNA Sequencer (Applied Biosystem, Forster City, CA, USA). All mutations were confirmed performing two independent PCR amplifications and their somatic origin was demonstrated, excluding the presence of the same mutation in the surrounding normal tissue.

### Drugs and reagents

Sorafenib (BAY-9006), provided by Bayer Pharmaceuticals Corporation, West Haven, CT, USA, was dissolved in Polyethylene Glycol 400 (PEG-400 Sigma-Aldrich, St. Louis, MO, USA) at a final concentration of 10 mM, and stored at -20°. The drug was diluted in RPMI 1640, (Invitrogen, Paisley, UK) to the desired concentration for *in vitro *studies. Vehicle was added to cultures as a solvent control. For *in vivo *experiments sorafenib tosylate was prepared fresh every day dissolving it in Cremophor EL (Sigma-Aldrich, St. Louis, MO, USA)/95% ethanol (50:50) following 20 minutes sonication. MEK-specific inhibitor UO126 (Promega Corporation, Madison, WI, USA) was prepared at an initial concentration of 10 mM in DMSO, stored at -80°C and used at a final concentration of 10 μM within 7 days. STI571 (purchased from Sequoia Research Products, Pangbourne UK) was stored in a 10 mM stock solution in dimethyl sulfoxide (DMSO) at -80°C.

### Cell growth assay

Cell viability was determined with Cell Titer-Glo^® ^luminescent cell viability kit (Promega Corporation, Madison, WI, USA) on OS cell lines after treatment with escalating doses of sorafenib (from 2.5 to 10 μM) at different time points (from 24 to 72 hours). This method is based on the mesurement of ATP production by cells, proportional to the number of viable cells, detected by luciferin-luciferase reaction. The luminescent signal developed was measured at 560 nm by DTX880 spectrofluorimeter multimode detection microplate reader (Beckam Coulter, Fullerton, CA, USA).

The IC_50 _value (concentration inhibiting 50% of the cell growth compared with PEG-400 control) and the relative confidential range were calculated for each cell line after 72 hours of sorafenib treatment using GraphPad Prism software version 5.0.

### DNA content analysis and detection of apoptosis

Following trypsinization, harvested cells were washed with chilled PBS. Cells were then fixed with 4 ml of chilled 70% ethanol and stored at -20°C. Following washing with chilled PBS, cells were pelleted and resuspended in 500 μl of PBS containing propidium iodide (PI, Sigma, 0.06 μg/ml) and RNase T1 (Roche, 1000 U/ml). Flow Cytometry was performed with FACS calibur employing the Cell Quest software. Cells with DNA content less than that of G0/G1-phase cells were considered to be apoptotic (sub-G0). Apoptosis was measured using the ApoAlert Annexin V-APC kit (Bender MedSystems Inc. Burlingame, CA). Cells were seeded in appropriate cell culture conditions in 60 mm plates. The following day, medium was replaced with fresh medium containing 10% FBS and the appropriate concentration of sorafenib. After 72 hours of incubation at 37°C, both adherent and non-adherent cells were harvested, washed once with cold PBS (0.15 mol/L, pH 7.2) and twice with binding buffer (150 mM NaCl_2_, 10 mM CaCl_2_, 10 mM Hepes). Cells were centrifuged at 3000 rpm for 5 min and resuspended in 1× binding buffer at a density of 1.0 × 10^6 ^cells per mL; 100 μL of the resuspended cells were incubated with APC conjugated annexin V and PI (0.5 μg/ml) for 15 min at RT in the dark. One hundred μL of 1 × binding buffer were added to the samples and the analysis was performed by FACS using Cell Quest Research Software (Becton Dickinson) and winMDI.2.8.

### Soft agar assay

Two thousand cells in 0.5 ml of 0.5% SeaPlaque Agarose low melting temperature (Lonza Rockland, ME, USA) with RPMI supplemented with 20% FBS and scalar concentrations of sorafenib were plated onto the top of the existing 1% bottom noble agar in each well of 24-well tissue culture plates. Plates were incubated at 37°C in a humidified atmosphere with 10% CO2 for three weeks. Medium was replaced with fresh medium and drug every 3 days. At the end of 3 weeks, colonies were stained with 0.05% crystal violet solution.

### CAM (chick chorioallantoic membrane) assay

Fertilized chicken embryos were incubated for 3 days at 37°C at 70% humidity. A small hole was made over the air sac at the end of the egg and a second hole was made directly over the embryonic blood vessels. After 7 days, cortisone acetate-treated filter disks (5 mm) were saturated with a) culture medium with 0,5% FBS; b) supernatant of 10^6 ^U2OS cells harvested after 72 h, c) same as b) plus 1 μM sorafenib and d) supernatant of U2OS cells treated for 72 h with 1 μM sorafenib. After 3 days CAMs were fixed with 4% parafolmaldehyde for 10 min at room temperature, filter disks were excised and pictures were taken with a QIcam FAST1394 digital color camera (QImaging) connected to the stereomicroscope (model SZX9; Olympus).

### Western Blot analysis

Five to ten million cells were washed with 1× PBS and lysed with lysis buffer (50 mM TrisHCl pH 7.5, 250 mM NaCl, 2 mM EDTA, 50 mM NaF, 0.1 mM Na_3_VO_4_, 0.5% Nonidet-P40, 1 mM DTT) and a cocktail of protease inhibitors (Sigma-Aldrich) for 15 minutes at 4°C and centrifuged at 14000 rpm for 15 minutes. The protein concentration of cell lysates was measured using the Bio-Rad DC Protein Assay kit (Bio-Rad Laboratories, Hercules, CA, USA) and 20 μg of proteins were resolved on 10% sodium dodecyl sulfate-polyacrylamide gel electrophoresis (SDS-PAGE) and electrotransferred to 0.45 μm PVDF (Amersham Pharmacia Biotech, Piscataway, NJ, USA) at 180 mA for 90 minutes at 4°C. Nonspecific sites were blocked by incubating for 1 hour with 5% non fat dry milk (Bio-Rad Laboratories, Hercules, CA, USA) in 1× Tris-buffered saline-Tween (TBST) (20 mM Tris-HCl pH 7.5, 500 mM NaCl, 0.1% Tween-20). Membranes were first incubated overnight with primary antibody and then with 1 μg/ml horseradish peroxidase conjugated secondary antibody (Amersham Pharmacia Biotech, Piscataway, NJ, USA) in 1× TBST with 1% non-fat dry milk. The anti-ERK1/2, P-ERK1/2 and MCL-1, P-ERM, ERM and β-actin were purchased from Cell signaling (Cell Signaling Technologies, Danvers, MA, USA). After each incubation step, membranes were washed 3 times for 15 minutes with 1× TBST, and revealed with a chemiluminescence reagent (LumiGLO, Cell Signaling Technologies, Danvers, MA, USA) and exposed to autoradiography film.

### ELISA assays

Sub-confluent OS cell lines were treated with sorafenib (5 μM) or PEG-400. The conditioned medium was collected 48 hours later, cleared by centrifugation at 14,000 rpm (4°C) for 5 minutes for ELISA analysis using Quantikine kits (R&D Systems, Minneapolis, MN) following manufacturer's instructions to quantify the amount of VEGF and metalloproteinase production. Cells were detached with trypsin and counted. All experiments were performed three times in triplicate and the mean and standard deviations were calculated. Results were done after normalization based on cell number.

### MCL-1 silencing

Validated small interfering RNAs (siRNA) targeting MCL-1 (SI02781205) and appropriate non-silencing control (AllStars Negative Control siRNA, 1027280) were synthesized by Qiagen. RNAi liposomes were generated using HyperFect Transfection Reagent (Qiagen) complexed with siRNA (final concentration 5 nM) in RPMI following the appropriate protocol. Samples were analyzed using Western Blot and FACS analysis at various time-points.

### Mice Xenograft models

CB.17 severe combined immunodeficient (SCID) female mice (5-6 weeks old; purchased from Charles Rivers (Lecco, Italy) were used for *in vivo *experiments. Animals were maintained at the animal facilities of CIOS (Torino, Italy) and handled according to institutional regulations, under sterile conditions in cage micro isolators. In three different experiments, one group of mice was injected subcutaneously (s.c.) into the right flank with 2.5 × 10^6 ^U20S in 50% growth factor-reduced BD Matrigel basement membrane matrix (BD Biosciences, San Jose, CA), and one group was injected intravenously (i.v.) with 10^5 ^SJSA-1. When the right flank xenografts were established at about 500 mm^3 ^and after 3 weeks for i.v. injected mice, the animals (5 mice per group) were treated daily with either sorafenib (10-30-100 mg/kg/die) or vehicle by oral gavage for 16 days and then sacrificed. S.c. xenograft diameters were measured every 7 days using calipers. Tumour volumes (V) were calculated using the following formula: V = A × B^2^/2 (A = largest diameter; B = smallest diameter). Lungs were examined macroscopically and microscopically for the presence of OS foci. For histological and immunohistochemical evaluations, lung and subcutaneous xenografts were collected and fixed in 10% formalin and embedded in paraffin. Sections of 4 μm-ticks were stained with Hematoxylin and Eosin and with Masson's trichromic staining for conventional histology. Patented blood vessels (vessels containing blood cells) were counted on Masson's trichromic stained s.c. xenograft sections from 3 different mice per group. The mean of identified patented vessels+/-standard deviation of 10 optical fields per slide were calculated. Lung foci were counted under optical microscope at 40× magnification on hematoxylin and eosin stained lung sections from 3 different mice per group. The surface occupied by OS cells was calculated as a percentage of the whole optical field.

### Statistics

Xenograft volume and immunohistochemical quantifications were analyzed using Student's T test. Categorical variables were analyzed using Fisher's exact test. P values < 0.05 were considered statistically significant.

## Competing interests

The authors declare that they have no competing interests.

## Authors' contributions

YP drafted the manuscript, designed the study, carried out the *in vitro *assays, the molecular studies and the *in vivo *experiments. GG and MA^1^conceived the study, helped in its design and drafted the manuscript. GC^1 ^carried out *in vivo *studies, drafted the manuscript and participated in the coordination of the study. MM and BT carried out immunohistochemical analysis. SB and GC^3 ^conceived, designed and performed the histochemical analysis on mice. MT participated in the molecular and immunohistochemical studies. AB carried out CAM assay. LG performed FACS analysis. GM participated in *in vivo *studies. FB supervised CAM assays; PP, FF, SF and MA^4 ^participated in the study and collected samples from patients. All authors read and approved the final manuscript.
